# Coexpression of HOXA6 and PBX2 promotes metastasis in gastric cancer

**DOI:** 10.18632/aging.202426

**Published:** 2021-02-01

**Authors:** Jianjiao Lin, Huiqiong Zhu, Linjie Hong, Weimei Tang, Jing Wang, Hongsong Hu, Xiaosheng Wu, Yaying Chen, Guangnan Liu, Qiong Yang, Jiaying Li, Yusi Wang, Zhizhao Lin, Yizhi Xiao, Weiyu Dai, Miaojvan Huang, Guoxin Li, Aimin Li, Jide Wang, Li Xiang, Side Liu

**Affiliations:** 1 Guangdong Provincial Key Laboratory of Gastroenterology, Department of Gastroenterology, Nanfang Hospital, Southern Medical University, Guangzhou 510515, China; 2 Department of Gastroenterology, Longgang District People’s Hospital, Shenzhen 518172, China; 3 The Second Affiliated Hospital of University of South China, Hengyang 421001, China; 4 Department of Gastroenterology, The Third Affiliated Hospital of Guangzhou Medical University, Guangzhou 510515, China; 5 Department of General Surgery, Nanfang Hospital, Southern Medical University, Guangzhou 510515, China

**Keywords:** HOXA6, PBX2, proliferation, metastasis, gastric cancer

## Abstract

HOXA6 gene plays a role of the oncogene in various cancers. Nonetheless, its effect on gastric cancer (GC) occurrence and development is still unclear. We analysed whether HOXA6 interacts with the PBX2 protein using the STRING database. The molecular mechanism by which HOXA6 synergizes with PBX2 in GC metastasis is not fully understood. Here, we found that the expression of HOXA6 was increased in GC tissues and cell lines. The upregulation of HOXA6 was closely associated with differentiation, lymph node metastasis, AJCC stage, TNM stage, and poor survival outcome in GC patients based on tissue microarray (TMA) data. Moreover, the overexpression of HOXA6 promoted, whereas siRNA-mediated repression of HOXA6 inhibited, the cell proliferation, migration, and invasion of GC cells. Furthermore, HOXA6 could physically interact with and stabilize PBX2. In addition, HOXA6 and PBX2 expression was positively correlated in GC cells and tissue. HOXA6 and PBX2 suppression in GC cells also led to decreased migration and invasion potential *in vitro*. *In vivo*, HOXA6 was shown to cooperate with PBX2 to enhance cell metastasis via orthotopic implantation. These data indicate that HOXA6 promotes cell proliferation, migration, and invasion and that the HOXA6-PBX2 axis may be a useful biomarker for disease progression in GC.

## INTRODUCTION

Gastric cancer (GC), a frequently common human cancer, ranks the 2^nd^ leading cause of cancer-associated mortality globally [1]. Currently, surgical resection has been identified as the possible treatment to cure GC, the advanced GC cases are associated with a low 5-year survival rate of about 5-20% and a short median overall survival (OS) of just 10 months [2–4]. GC patients have dismal prognostic outcomes, which may be attributed to the high distant metastasis (DM) and tumor relapse rates following curative surgery. But it is still unknown about the precise mechanism of GC metastasis at molecular level. As a result, it is of vital importance to identify new molecular markers, so as to prevent GC metastasis and relapse.

The homeobox (HOX) gene family display some degree of similarity to a short 180 bp motif and exert a vital part in the normal biological processes as well as carcinogenesis [5–7]. As key transcription factors, encoded homeodomain proteins can bind to the unique DNA sequences in the form of monomers or multimers, thus regulating cell proliferation, differentiation or apoptosis [6]. There are 39 HOX genes discovered in human autosomal chromosomes, which are classified as four paralogous clusters (namely, HOXA, HOXB, HOXC and HOXD) [7]. Genes in the HOX family have been previously reported to participate in carcinogenesis and are related to prognosis [8–10]. Typically, the over-expression of HOXA13, HOXB5, HOXC6 and HOXD4 are detected in GC, which is found to promote GC cell proliferation [11–14].

HOXA6 belongs to HOX family [15, 16]. Some evidence shows that HOXA6 genes are deregulated in multiple cancers, such as cervical cancer [17], cerebral glioma [18], renal cancer [19] and colorectal cancer (CRC) [20]. For example, HOXA6 may promote the proliferation, migration and invasion of CRC cells, as shown using a series of experiments in CRC cells [20]. Additionally, the expression of HOXA6 may be regulated by miR-1294 in clear cell renal cell carcinoma [21]. However, the relationship of HOXA6 level with GC development remains unclear so far.

PBX2 protein belongs to PBX family, and it shows ubiquitous expression in a variety of tissues [22, 23]. PBX2 plays a role of a cofactor, which regulates numerous gene expression levels together with other proteins and induces cellular function execution, including suppression of cell proliferation, differentiation and apoptosis [24–26]. PBX2 genes are found to be abnormally expressed in different cancer types, including esophageal squamous cell carcinoma (ESCC), gastric carcinoma (GC) [25], gingival squamous cell carcinoma [27], along with non-small cell lung carcinoma (NSCLC) [28]. PBX2 knockdown is discovered to down-regulate the colony formation ability *in vitro* and the tumorigenicity *in vivo* within cancer cells. Additionally, the up-regulated PBX2 level is identified as the factor to independently predict the poor prognosis of ESCC and GC, and PBX2 may suppress cell apoptosis to enhance tumor development [25]. We analyzed the STRING database and showed that HOXA6 may interact with the PBX2 protein. However, the exact molecular mechanisms by which HOXA6 synergizes with PBX2 in GC cells remain elusive.

In the present study, we detected HOXA6 expression and investigated the biological functions of HOXA6 in GC as well as the potential prognostic significance of HOXA6 expression in GC. Moreover, the present work was conducted aiming to explore the association of HOXA6 with PBX2 within GC as well as the impact of HOXA6 co-expressed with PBX2 on GC cell invasion and migration. Our study provides information for the targeted therapy of GC.

## RESULTS

### HOXA6 expression is upregulated in human GC tissues and predicted unfavorable prognosis in GC patients

**Figure 1:**
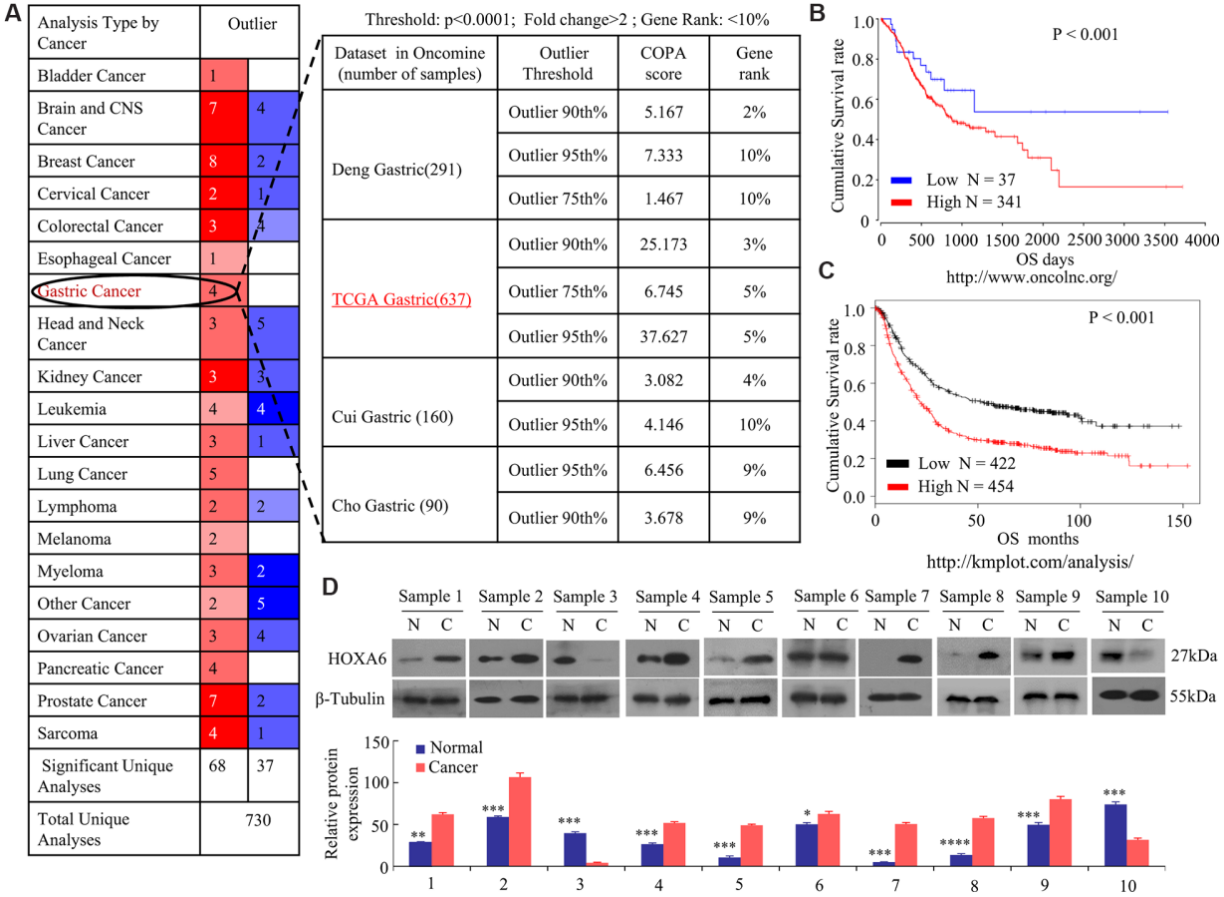
**Increased levels of HOXA6 in GC data sets from public databases and human GC tissue.** (**A**) Analysis of four Oncomine datasets showed that the mRNA expression levels of HOXA6 in GC are significantly higher than in normal tissues. The numbers in the red boxes in the Outlier panel in the table denote the number of data sets. (**B**, **C**) Kaplan-Meier plots from data on the OS of patient cohorts from the OncoLnc (**B**) and Plotter (**C**) databases. N = number; OS, overall survival. (**D**) The relative HOXA6 protein expression levels in 10 pairs of GC tissues (**C**) and matched adjacent non-tumorous tissues (N). b-Tubulin expression was measured and served as the loading control. The grey level of each band quantified. The protein expression relative levels were compared with Quantity One software. *, P > 0.05; **, P < 0.05; ***, P < 0.01; ****, P < 0.001.

In order to ascertain the significance of HOXA6 in gastric cancer, we first analyzed the expression of HOXA6 in GC with multiple cancer microarray data sets available from Oncomine (https://www.oncomine.com/). As shown in Figure 1A, HOXA6 mRNA levels were upregulated in 4/4 (100%) GC data sets. Notably, the expression of HOXA6 in gastric cancer tissues were clearly higher than those in matched normal nontumorous tissues in a large sample dataset from the TCGA database (637 samples). Kaplan-Meier survival analyses of data from the OncoLnc (http://www.oncolnc.org/) and KMPlotter (http://kmplot.com/analysis/) databases revealed that cases who had increased HOXA6 level showed reduced overall survival (OS) relative to cases who had decreased HOXA6 level (Figure 1B, 1C). We then observed that 8/10 pairs of human GC tissues showed markedly increased HOXA6 expression compared with their matched controls using Western blot analysis (Figure 1D).

**Figure 2:**
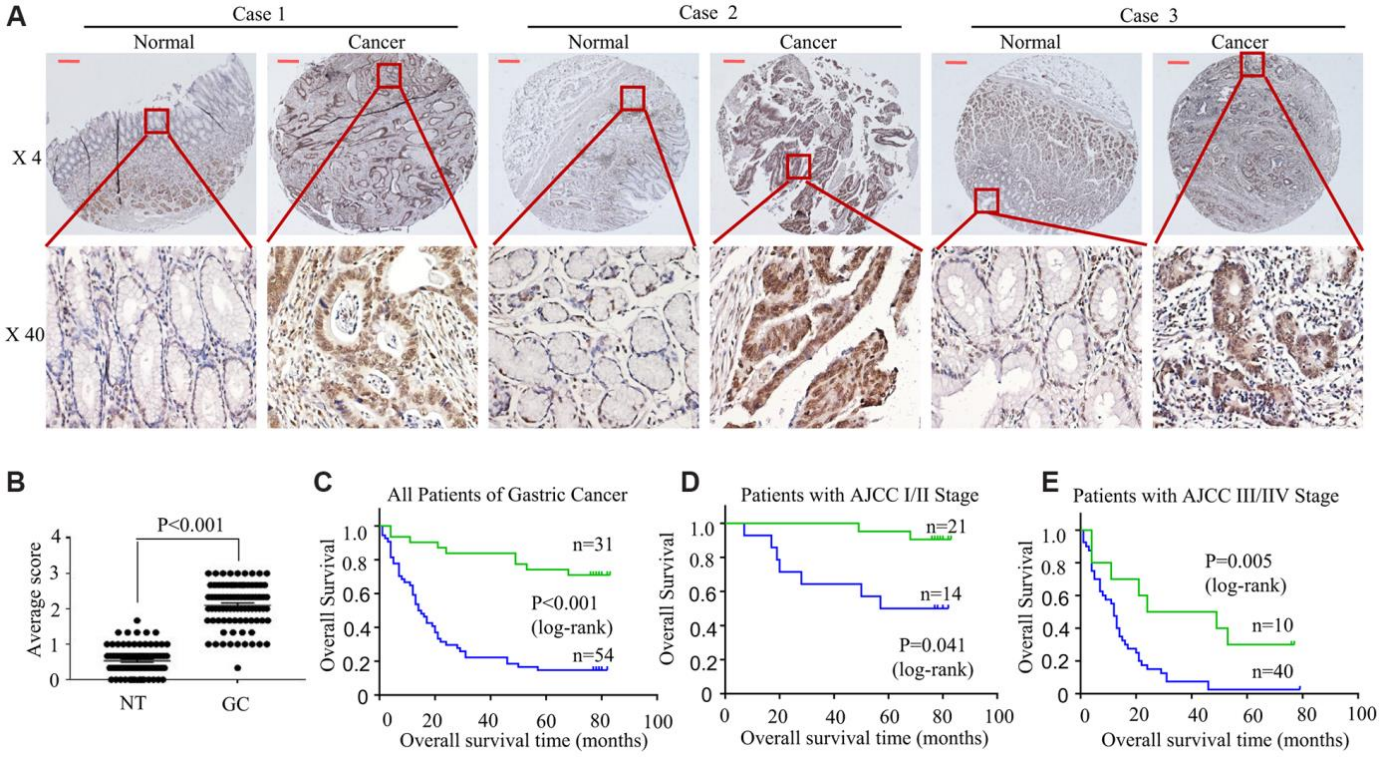
**HOXA6 is highly expressed in GC tissues predicted unfavourable prognosis in GC patients.** (**A**) We detected HOXA6 expression in GC tissues and adjacent normal tissues by TMAs. Representative views are shown. (**B**) HOXA6 protein scores in NT and GC as detected using a TMA-based assay. NT, normal tissue; GC, gastric cancer (**C**) The overall survival times of 85 patients with GC categorized as “low HOXA6” or “high HOXA6” groups after surgery are compared. (**D**, **E**) HOXA6 expression in patients with early-stage GC (**D**) and late-stage GC (**E**). Log-rank test was employed to calculate the statistical significance. Scale bars in A represent 25 μm.

For better understanding how HOXA6 level affected GC genesis and development, we performed an immunohistochemical analysis with tissue microarray (TMA) of 85 gastric tissue cases. Relative to normal gastric tissue samples, up-regulated HOXA6 protein level was detected within GC tissue samples, as shown in Figure 2A. Semiquantitative scoring showed that HOXA6 protein level increased within GC tissue samples relative to matched non-carcinoma samples (Figure 2B).

For better analyzing the association of HOXA6 level with patient clinicopathological features and prognosis, we classified all cases to two groups, including the high or low HOXA6 expression group, according to the median HOXA6 level. It was illustrated from Table 1 that, HOXA6 level showed significant correlation with tumor size (< 5cm versus (vs) ≥ 5cm, P =0.001), differentiation (P =0.002), lymph node metastasis (P < 0.001), TNM (I/II vs. III/IV, P < 0.001), AJCC (I/II vs. III/IV, P < 0.001). There was no statistical significance among HOXA6 expression and other features, including gender and age (<60 y vs. >60 y) (all, P >0.05).

**Table 1:** Correlation between HOXA6 protein expression and the clinicopathological parameters of gastric carcinoma.

**Features**	**Total number (n =85)**	**HOXA6 expression**		**P**
		**Low**	**High**	
Age (years)				0.062
<60	33	8(24.2%)	25(75.8%)	
≥60	52	23(44.2%)	29(55.8%)	
Gender				0.347
Male	52	21(40.4%)	31(59.6%)	
Female	33	10(30.3%)	23(69.7%)	
Differentiation				0.002
Well	21	14(66.7%)	7(33.3%)	
Moderate	26	9(34.6%)	17(65.4%)	
Poor	38	8(21.1%)	30(78.9%)	
Lymph node metastasis				<0.001
Yes	63	15(23.8%)	48(76.2%)	
No	22	16(72.7%)	6(27.3%)	
Tumor size (cm3)				<0.001
<5	15	10(66.7%)	5(33.3%)	
≥5	70	21(30.0%)	49(70.0%)	
AJCC T stage				<0.001
T1, T2	13	12(92.3%)	1(7.7%)	
T3, T4	72	19(36.5%)	53(63.5%)	
AJCC TNM stage				<0.001
I, II	35	21(60.0%)	14(40.0%)	
III, IV	50	10(20.0%)	40(80.0%)	

Furthermore, upregulated HOXA6 expression was strongly associated with shortened overall patient survival based on TMA stage (Figure 2C). In addition, patients were stratified into two subgroups according to the AJCC and TNM data (I/II vs. III/IV), and III/IV stage (Figure 2E) patients with higher HOXA6 expression had a poorer prognosis than early-stage patients (I/II, Figure 2D).

In summary, our data suggest that the aberrant overexpression of HOXA6 in GC predicted unfavorable prognosis in GC patients

### Overexpression of HOXA6 enhances the proliferation, migration, and invasion of gastric cancer cells

**Figure 3:**
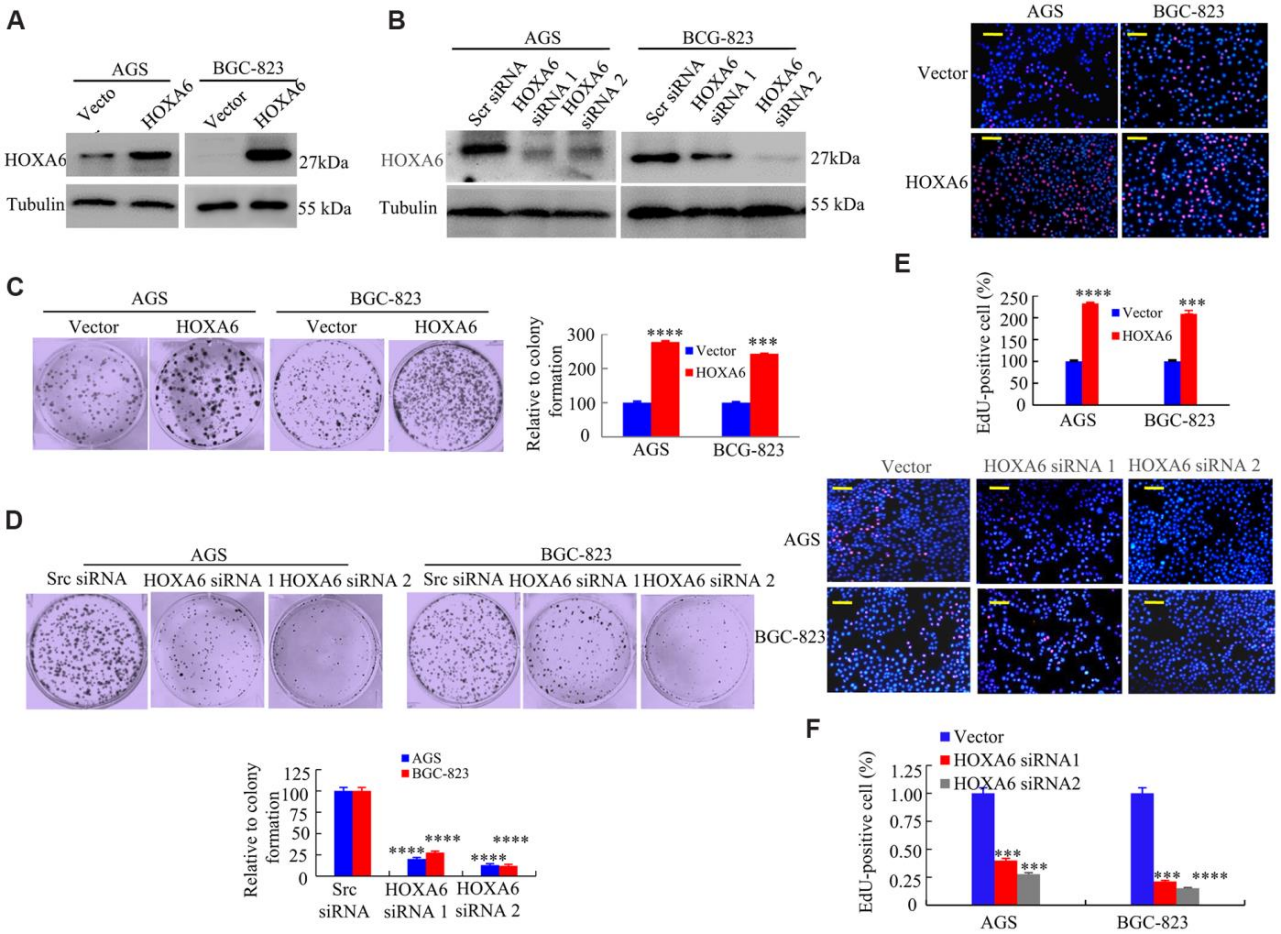
**The effects of HOXA6 on proliferation in GC cells.** (**A**, **B**) HOXA6 protein levels in HOXA6-overexpressing (**A**) or HOXA6 knockdown (**B**) measured the GC cell lines by Western blotting assay. b-Tubulin was used as a control. (**C**, **D**) Representative images from a colony formation assay with AGS and BGC-823 cells. Quantified colony numbers. Data are shown as the mean (n = 3) ± SD. ***, P < 0.01; ****, P < 0.001. (**E**, **F**) EdU incorporation assay in GC cells following transfection with HOXA6 plasmid or HOXA6 siRNA. EdU-positive cells was indicated by red fluorescence, and the total cells was indicated blue fluorescence from Hoechst stain. ***, P < 0.01; ****, P < 0.001. Scale bars in E and F represent 100 μm.

HOXA6 is up-regulated in various cancers [17, 20], which suggests that the over-expressed HOXA6 level may promote GC development. To test this hypothesis, we attempted to identify the biological function of HOXA6 in AGS and BGC-823 cells by overexpression and knockdown with siRNA (Figure 3A, 3B). As shown in Figure 3C–3F, the overexpression of HOXA6 promoted proliferation, while proliferation was significantly inhibited following the knockdown of HOXA6 compared with control AGS and BCG-823 cells, as determined using clone formation and EdU assays.

**Figure 4:**
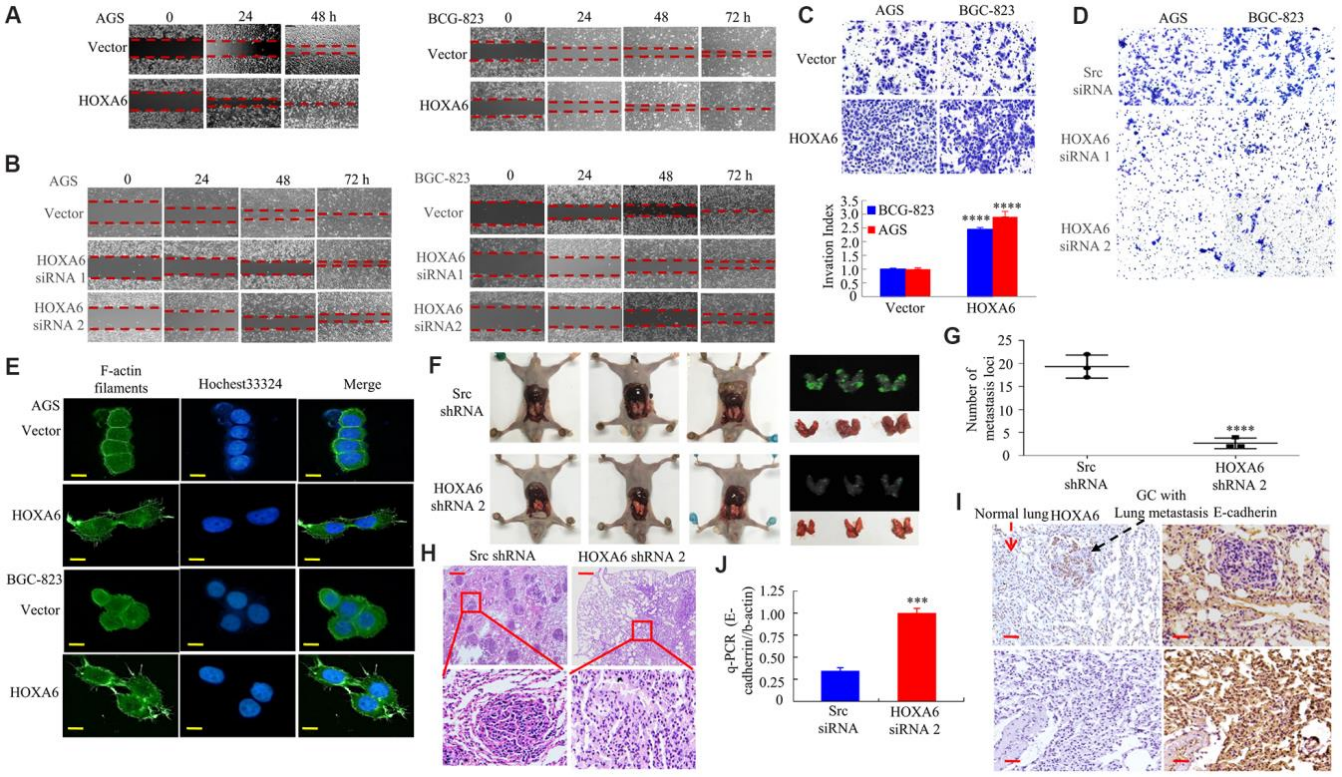
***In vitro* and *in vivo*, the effects of HOXA6 on migration and invasion in GC cells.** (**A**, **B**) Wound healing assay in GC cells transfected with HOXA6 plasmid or HOXA6 siRNA. Relative wound closure was calculated in experiments performed in triplicate. (**C**, **D**) Transwell assay conducted in GC cells transfected with HOXA6 plasmid or HOXA6 siRNA. The number of invading cells is shown. Cells in 5 independent symmetrical visual fields from three independent experiments were counted under a microscope. ****, P < 0.001. (**E**) Stable HOXA6 transfectants were stained with rhodamine-phallotoxin to reveal F-actin filaments by fluorescence microscopy. (**F**) Whole-body fluorescence imaging showing GC progression in mice (n = 3). Metastatic loci in the lungs are detected in the image. (**G**) Metastatic loci in the lung were calculated. ****, P < 0.001. (**H**) Lung sections were stained with haematoxylin and eosin. (**I**) The expression of HOXA6 and E-cadherin in the lung metastasis in GC was determined by IHC. (**J**) qRT-PCR was detected the expression of E-cadherin in lung tumours derived from AGS cells. ***, P < 0.01. Scale bars, 50 μm in E, 100 μm in **H** and **I**.

Later, this study examined how HOXA6 affected the invasion and migration of cells. As shown in Figure 4A–4D and Supplementary Figure 1A–1C, the ectopic expression of HOXA6 increased cell migration and invasion, whereas HOXA6 knockdown had the opposite effect on AGS and BCG-823 cells.

A report showed that the *homeobox gene* HOXB7 promotes F-actin polymerization and migration in cancer cells [29]. Therefore, we then transfected GC cells with HOXA6 or a vector, followed by staining for F-actin using phalloidin. Relative to cells that expressed vector, F-actin fibers were detected in cells with HOXA6 over-expression, which participated in cancer cell metastasis and invasion that were concentrated mainly just inside the cytoplasm and margin (Figure 4E).

For better exploring the effect of HOXA6 on tumor metastasis *in vivo*, we adopted the AGS cell pulmonary metastasis model in the present work. AGS GC cells were infected with Lenti-HOXA6 shRNA 2 or Lenti-Src shRNA. Western blot analysis verified the knockdown of human HOXA6 in AGS/HOXA6 cells (data not shown).

HOXA6 shRNA 2 or Src shRNA-transfected cells were injected via the tail vein into syngeneic BALB/Cnu/nu nude mice. Four weeks later (Figure 4F), lung tissues were harvested and the number of metastatic colonies was calculated. Pulmonary metastases injected with HOXA6 shRNA 2-transfected cells was decreased compared to Src shRNA-transfected cells in mice (Figure 4G). Histological analysis further verified GC lung metastasis (Figure 4H). Loss of E-cadherin promotes metastasis in multiple cancers [20, 29]. Moreover, HOXA6 was up-regulated while E-cadherin was down-regulated within cancer tissues relative to non-carcinoma samples of nude mice (Figure 4I, 4J), as demonstrated by IHC and q-PCR assays.

These data showed that HOXA6 promotes malignant biological behavior in GC cells.

### HOXA6 physically interacts with PBX2 in GC cells

**Figure 5:**
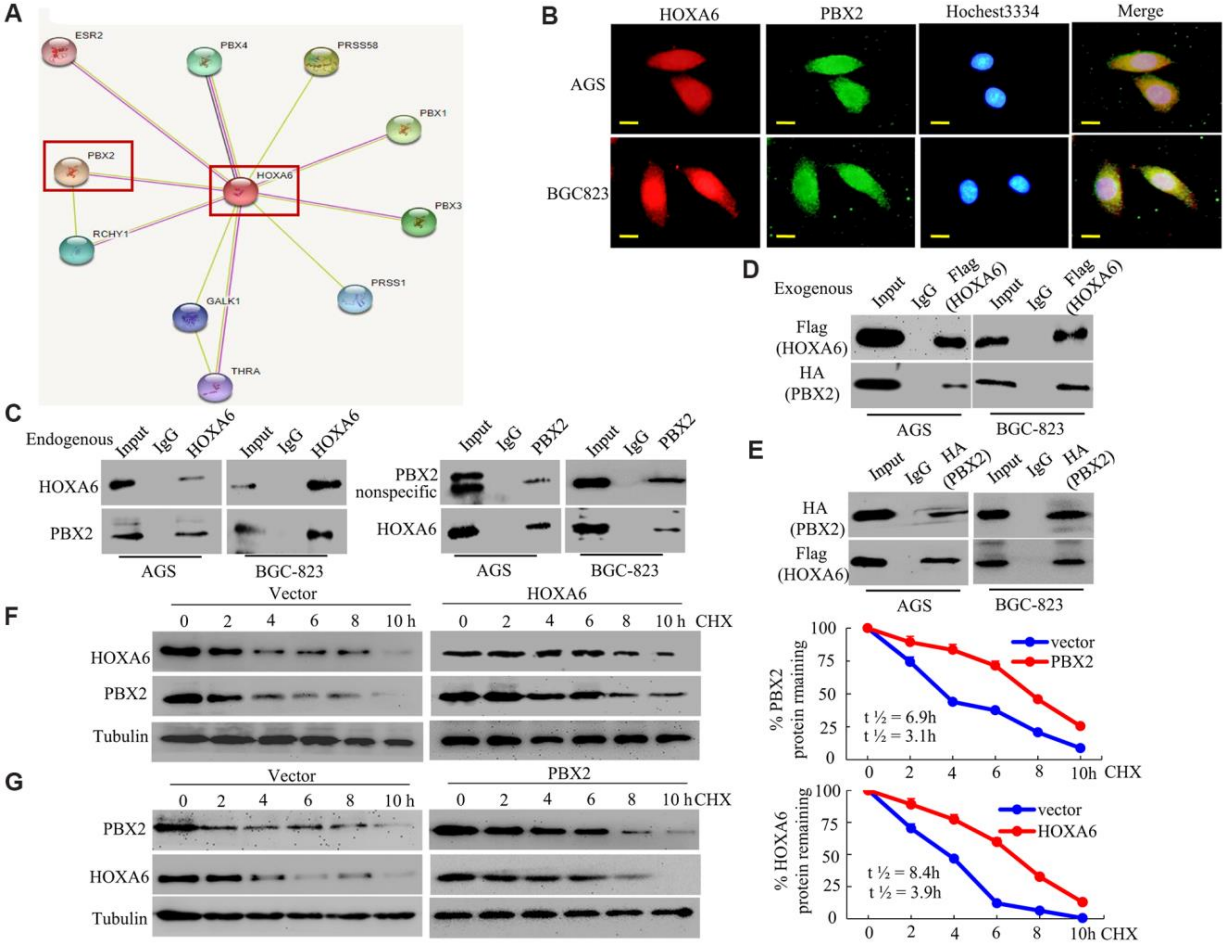
**HOXA6 physically interacts with PBX2 in GC cells.** (**A**) HOXA6-related protein-protein interaction (PPI) network from the STRING database. (**B**) Confocal microscopy detected the interaction of HOXA6 and PBX2 in GC AGS and BCG-823 cells. HOXA6 and PBX2 colocalization was observed in the nuclear, perinuclear and cytoplasmic regions of both cell lines. (**C**) To conduct immunoprecipitation with anti-HOXA6 (PBX2) antibody, either normal mouse IgG (nmIgG) or anti-HOXA6 (PBX2) was immunoprecipitated from whole-cell lysate antibody, followed by western blotting with anti-PBX2 (HOXA6) antibody. (**D**) HOXA6-Flag was co-transfected with PBX2-HA into AGS and BCG-823 cells. Whole-cell extracts were immunoprecipitated with an anti-Flag antibody and blotted with an anti-HA antibody. (**E**) PBX2-HA was co-transfected with HOXA6-Flag into AGS and BCG-823 cells. Anti-HA antibody was performed with immunoprecipitation, and pre-immune normal mouse immunoglobulin G (nm IgG) was designated as a reference control. Western blot analysis was used for anti-Flag antibody. (**F**, **G**) Regulation of PBX2 (HOXA6) protein stability by HOXA6 (PBX2). 50 μg/mL cycloheximide (CHX) was added to vector-transfected AGS cells overexpressing HOXA6 (PBX2) after the start of the experiment. The cells were harvested at the indicated times and lysates were prepared after the addition of cycloheximide. Western blotting was used to perform. Scale bars in B represent 25 μm.

To better understand the function of HOXA6, we analyzed the interactions between HOXA6 and other proteins using the STRING database (online database resource Search Tool for the Retrieval of Interacting Genes). The results revealed that HOXA6 potentially interacts with 10 proteins: ERR2, PRSS1, PRSS58, Galk1, THRA, RCHY1, PBX1 (Pre B cell leukemia homeobox), PBX3, PBX4, and PBX2 (Figure 5A). Among them, PBX1, PBX2, PBX3 and PBX4, which are members of the PBX gene family, may be involved in tumor cell development [25, 30–32]. PBX1, PBX3, and PBX4 regulate the proliferation and differentiation of GC cells [30, 31], and some studies have shown that HOXA6 may be related to the PBX1 or PBX3 proteins [33, 34]. However, more studies are needed to determine how HOXA6 and PBX2 affect GC genesis and development.

First, we analyzed the intracellular colocalization of HOXA6 and PBX2 in AGS and BCG-823 cells by confocal microscopy. Endogenous HOXA6 and PBX2 mostly concentrated in GC cell nuclei, and the merged signal indicated colocalization of the two proteins (Figure 5B).

Second, to determine whether HOXA6 interacts with PBX2 *in vivo*, we performed coimmunoprecipitation (Co-IP) and Western blot analyses using anti-HOXA6 and anti-PBX2 antibodies. As shown in Figure 5C, endogenous HOXA6 and PBX2 proteins interacted in the lysates of GC cells. Third, we cotransfected AGS and BCG-823 cells with expression plasmids encoding Flag-tagged HOXA6 and HA-tagged PBX2. The exogenous HOXA6 and PBX2 proteins were shown to be associated in AGS and BCG-823 cells using reciprocal Co-IP experiments (Figure 5D, 5E).

Fourth, to address whether HOXA6 and PBX2 interact and mutually stabilize each other, AGS cells were transfected with HOXA6 plasmid or vector, followed by incubation with cycloheximide to block new protein production. At diverse time points after cycloheximide was added, cellular extracts were made, and Western blotting assay was conducted to measure PBX2 protein level. It was shown in Figure 5F that, the overexpression of HOXA6 reduced the rate of PBX2 degradation in the cells. Quantification of PBX2 levels revealed a statistically significant difference in PBX2 expression between CHX-treated HOXA6-overexpressing cells and vector-treated cells. For the endogenous PBX2, its half-life elevated from 3.1 h to about 6.9 h after HOXA6 over-expression. Consistently, PBX2 increased the stability of the HOXA6 protein in PBX2-overexpressing GC cells (Figure 5G).

In conclusion, the above findings suggested the interaction of PBX2 with HOXA6 and the mutual stabilization of them with exogenous and endogenous proteins in GC cells.

### HOXA6 expression positively correlates with PBX2 expression in GC

**Figure 6:**
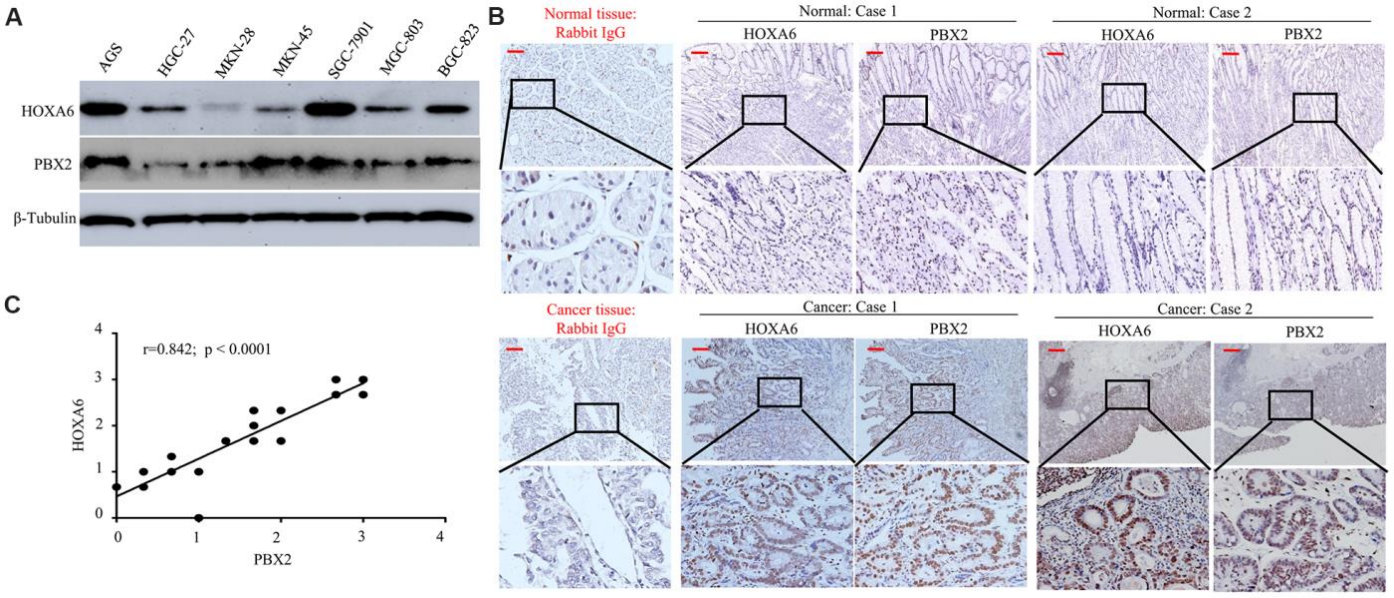
**The positive expression of HOXA6 correlated with PBX2 expression.** (**A**) Western blot analysis was is used to determinate the expression of HOXA6 and PBX2 in GC cell lines. (**B**) Representative IHC images of HOXA6 and PBX2 in GC (n =23) and their matched adjacent tissues following serial sectioning. The first antibody used for normal rabbit IgG as an isotype control. (**C**) Spearman correlation analysis of HOXA6 and PBX2 protein levels in GC tissue. Scale bars in B represent 50 μm.

For determining the value of HOXA6 and PBX2 levels clinically, we performed Western blotting experiments on the HGC-27, AGS, MKN-45, MKN-28, SGC-7901, BGC-823 and MGC-803 GC cell lines. It was discovered that HOXA6 level was positively associated with PBX2 for six of the above seven cell lines but not in MKN-45 cells (Figure 6A).

Next, we detected the expression of HOXA6 and PBX2 in 23 gastric mucosa tumor tissue specimens and adjacent nontumor tissue by IHC staining. Expression of both the HOXA6 and PBX2 proteins was high in cancer cells but either absent or extremely low in normal tissues, as shown in Figure 6A, 6B. Pearson correlation analysis showed that HOXA6 level showed positive association with PBX2 level within cancer tissues (Figure 6C).

These experiments suggest that the positively expression of HOXA6 is associated with PBX2 expression in gastric cancer.

### HOXA6 and PBX2 promote the migration and invasion of GC cells *in vitro* and *in vivo*

**Figure 7:**
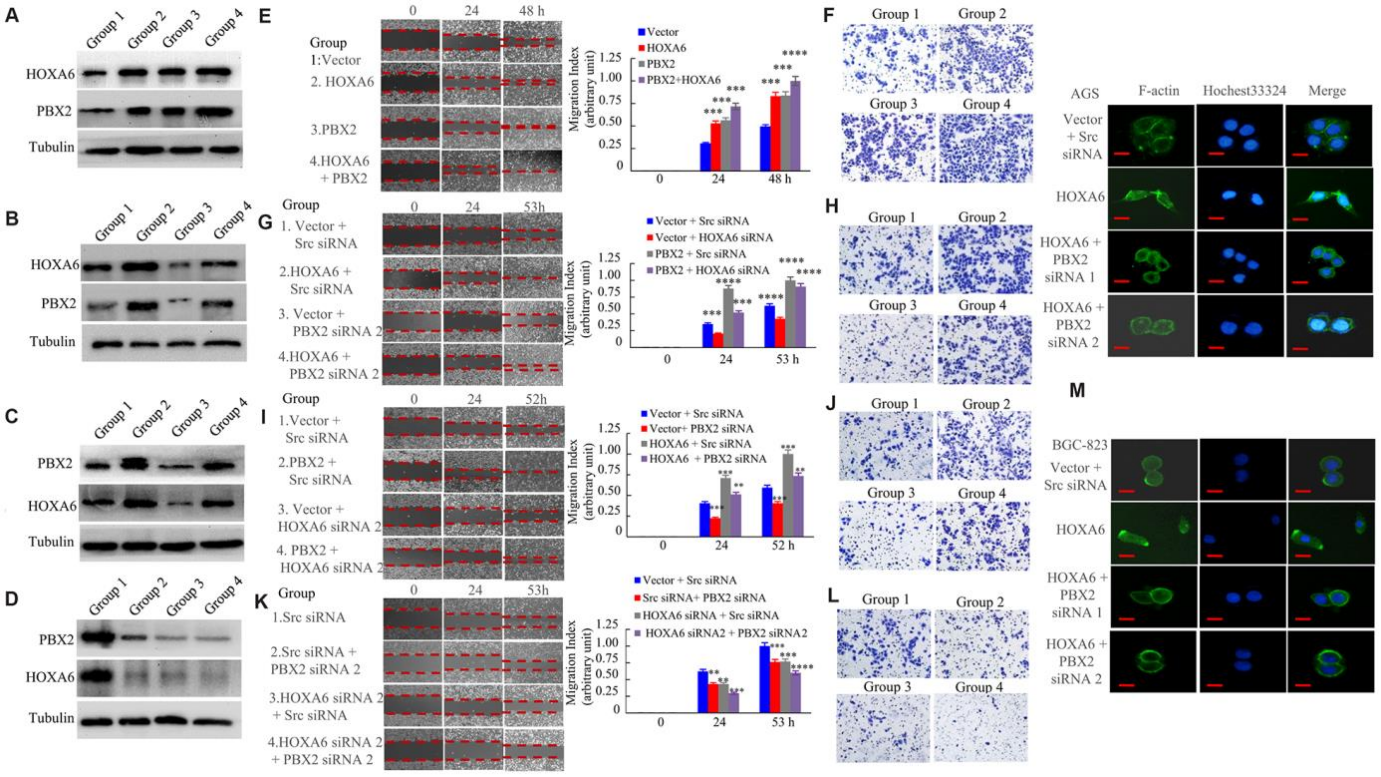
**HOXA6 and PBX2 promote the migration and invasion of gastric cancer cells.** (**A**) The expression of vector (group 1), or HOXA6 (group 2), or PBX2 (group 3), or HOXA6 plus PBX2 (group 4) in AGS stable transfectants cells detecting by western blot assay. (**B**) The expression of vector plus scrambled siRNA (Scr siRNA, group 1), or HOXA6 plus Scr siRNA (group 2), or vector plus PBX2 siRNA 2 (group 3), or HOXA6 plus PBX2 siRNA 2 (Group 4) in AGS transfectants cells at protein level as detected. (**C**) The expression of vector plus Scr siRNA (group 1), or PBX2 plus Scr siRNA (group 2), or vector plus HOXA6 siRNA 2 (group 3), or PBX2 plus HOXA6 siRNA 2 (Group 4) in AGS transfectants cells measuring by western blot analysis. (**D**) The expression of Scr siRNA (group 1), or Src siRNA plus PBX2 siRNA 2 (group 2), or HOXA6 siRNA 2 plus Src siRNA (group 3), or HOXA6 siRNA 2 + PBX2 siRNA 2 was subjected to western blot analysis. (**E**–**L**) GC cells were evaluated by wound healing and transwell invasion assays. Photographs show cell migration into the wounded area, and the data shows the percentage of wound closure in the histogram. Photographs show the cells that travelled through the micropore membrane. **, p < 0.05; ***, p < 0.01; ****, p < 0.001. (**M**) The F-actin filaments of GC cells staining with rhodamine-phallotoxin for 48h were observed by fluorescence microscopy. Scale bars in M represent 20 μm.

For determining the synergistic effect of HOXA6 and PBX2 on promoting gastric cancer cell invasion and migration, we examined the effect of HOXA6 or PBX2 overexpression or knockdown with siRNA. First, ectopic HOXA6 or PBX2 expression or knockdown with siRNA in GC cells was confirmed by Western blot assays (Figure 7A–7D). Then, we determined that the forced expression of HOXA6 and PBX2 significantly increased wound healing and cell migration in comparison with cells expressing HOXA6 or PBX2 alone (Figure 7E), and the relative distance migrated by the cells was significantly longer (Figure 7E). Moreover, an invasion assay was applied to quantify the invasive ability of AGS cells expressing HOXA6 or PBX2. Obviously, cotransfection with plasmids encoding HOXA6 and PBX2 significantly enhanced AGS cell invasion after 24 h compared with that in cells expressing HOXA6 or PBX2 alone, and the relative number of invading cells was remarkably higher (Figure 7F and Supplementary Figure 2A).

Furthermore, we sought to test the effects of HOXA6 and PBX2 on migration and invasion by RNA interference. AGS cells transfected with HOXA6 illustrated enhanced cell migration and invasion in comparison with vector-transfected cells, whereas the siRNA-mediated knockdown of PBX2 in AGS cells led to a decreased migration (Figure 7G) and invasion (Figure 7H and Supplementary Figure 2B) potential of PBX2 siRNA in HOXA6-overexpressing cells. Similarly, the transient transfection of PBX2 increased AGS cell migration (Figure 7I) and invasion (Figure 7J and Supplementary Figure 2C), while HOXA6 knockdown by treatment with HOXA6 siRNA inhibited the migration and invasion capacity of PBX2-overexpressing AGS cells.

In addition, we treated GC cells with HOXA6 siRNA or PBX2 siRNA alone, which impaired migration and invasion. In addition, treatment with HOXA6 siRNA and PBX2 siRNA decreased cell migration (Figure 7K) and invasion more than treatment with either siRNA in AGS cells (Figure 7L and Supplementary Figure 2D).

The abovementioned results show that the overexpression of HOXA6 promotes F-actin filament assembly. However, the depletion of PBX2 in HOXA6-overexpressing cells caused the formation of weak and aggregated F-actin filaments (Figure 7M).

**Figure 8:**
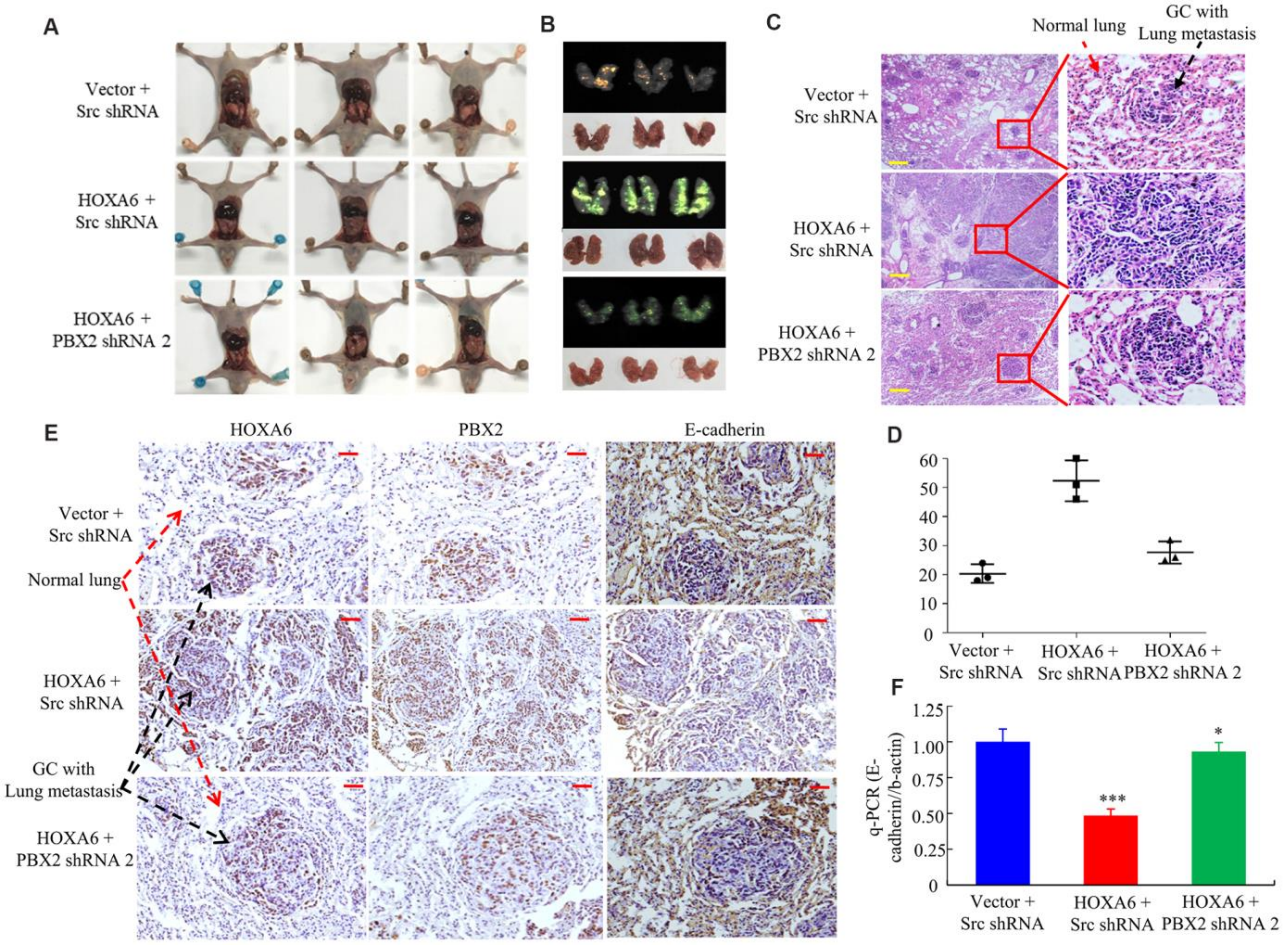
***In vivo*, HOXA6 synergizes with PBX2 to accelerate tumour metastasis.** (**A**, **B**) All mice were sacrificed 4 weeks after tail vein injection of vector, HOXA6 + Src shRNA or HOXA6 + PBX2 shRNA 2. (**A**) and whole-body white light images. (**B**) orthotopic tumours from mice lung metastases. (**C**) Haematoxylin and eosin (H&E) staining of lungs was performed in samples from mice. (**D**) Metastatic lesions in the lung were counted. (**E**) The expression of HOXA6, PBX2 and E-cadherin in the lung metastasis of GC was detected through immunohistochemistry method. (**F**) E-cadherin expression in tumours derived from AGS cells was detected by qPCR. ***, P < 0.01, vector versus HOXA6; HOXA6 versus HOXA6-PBX2 shRNA; *, P > 0.05, vector versus HOXA6-PBX2 shRNA. Scale bars in C and E represent 100 μm.

For understanding the synergistic effect of HOXA6 and PBX2 on promoting tumor metastasis *in vivo*, we injected cells transfected with AGS/Lenti-vector, AGS/Lenti-HOXA6 or AGS/Lenti-HOXA6-PBX2 shRNA that expressed green fluorescent protein (GFP) into nude mice via tail vein injection to examine lung metastasis. Up-regulated HOXA6 level led to a higher lung metastasis rate and more metastatic lung nodules in comparison with vector-transfected cell injection group, while PBX2 knockdown in cells over-expressing HOXA6 resulted in a lower lung metastasis rate and less metastatic loci (Figure 8A, 8D). Moreover, an IHC assay showed that the HOXA6 and PBX2 proteins were expressed at high levels, whereas the E-cadherin protein was expressed at a low level in the xenograft tumors (Figure 8E).

According to q-PCR analysis, HOXA6 induction led to marked down-regulation of E-cadherin, while the down-regulated PBX2 level cells over-expressing HOXA6 might up-regulated E-cadherin protein level (Figure 8F).

Taken together, these results indicated that HOXA6 and PBX2 mutually and significantly promoted GC cell migration and invasion.

## DISCUSSION

Our present work first analyzed the role HOXA6 in GC and exhibited that HOXA6 was upregulated in human gastric cancer clinical specimens in comparison to normal gastric tissues. The up-regulated HOXA6 level was associated with dismal GC prognosis. Moreover, GC cells in which HOXA6 was upregulated tended to display more aggressive phenotypes. In addition, we report that HOXA6 interacts with PBX2 and that the coexpression of HOXA6 and PBX2 is positively associated with migration and invasion in GC cells. According to the above findings, HOXA6-PBX2 axis exerts a vital part in GC.

HOXA6 belong to the HOX protein family, and the importance of the HOXA cluster in carcinogenesis has been highlighted. High HOXA6 expression is previously discovered in some tumors, for instance, cervical cancer and colorectal cancer [17, 20]. Moreover, HOXA6 was upregulated in patients with acute myeloid leukemia [35]. Nonetheless, it is still unclear about the HOXA6 pathogenic mechanism in GC. This work high HOXA6 levels in most of gastric cancer tissues compared to matched normal gastric tissues by tissue microarray. Moreover, HOXA6 expression was associated with adverse clinicopathological parameters. Ectopic expression of HOXA6 promoted the growth, clone formation, migration and invasion capacity of gastric cancer cells, as shown by gain- and loss-of-function experiments. This finding is consistent with the finding in previous studies that the overexpression of HOXA6 promotes CRC cell invasion and metastasis [20]. In addition, Eoh KJ et al. reported that overall survival was significantly shorter in cervical cancer patients with high expression of HOXA than in those with low expression of HOXA [17]. Similarly, GC patients whose tumors had high HOXA6 levels exhibited significantly worse overall survival, indicating that a high level of the HOXA6 protein in tumors is a marker of poor prognosis in GC patients. The above results indicated the up-regulation of HOXA6 within GC, which exerted a critical part in GC genesis and development.

The regulatory mechanism of HOXA6 overexpression in human GC remains unknown. Using bioinformatics approaches, we showed that the HOXA6 protein may interact with PBX2 to carry out its function. PBX2, a transcription factor, is located on chromosome 6p21 [36] and has been reported to be expressed strongly in the nuclei of GC cells [25]. PBX2 is postulated to play an important part in the upregulation of tumor-correlation protein expression [37, 38]. Here, the subcellular distribution of the HOXA6 and PBX2 proteins in GC cells demonstrated their colocalization. Moreover, both endogenous and exogenous HOXA6 and PBX2 were associated in GC cells, as determined using reciprocal Co-IP experiments. This observation is consistent with the previous finding that the homeobox (HOX) gene HOXA13 associates with MEIS1B in mammalian and yeast cells and that HOXA13 can interact with all MEIS proteins [39]. Furthermore, the HOXA6 and PBX2 proteins interact and mutually stabilize each other in GC cells. In addition, HOXA6 and PBX2 levels were markedly up-regulated within GC samples compared with the matched non-carcinoma samples. Our results reveal that the HOXA6-PBX2 signaling axis may regulate GC cell function.

HOXA6 played a crucial part in tumor metastasis and invasion, we questioned whether HOXA6 synergizes with PBX2 to promote GC metastasis. The present data indicated that overexpressing HOXA6 further increased the PBX2 overexpression-induced increase in GC cell invasion and migration, whereas the ectopic expression of HOXA6 in cells treated with PBX2 siRNA or PBX2 in cells treated with HOXA6 siRNA inhibited cell migration and invasion. Besides, HOXA6 and PBX2 knockdown evidently suppressed the invasion and migration of cells compared to the knockdown of HOXA6 or PBX2 alone. Hence, the data illustrated that HOXA6 co-expressed with PBX2 enhanced GC cell metastasis and invasion.

In conclusion, we report a novel function of HOXA6 in GC growth and metastasis. HOXA6 expression increases within GC samples, which is related to GC progression as well as the dismal prognostic outcome. Moreover, HOXA6 boosts gastric cancer cell migration, invasion, and proliferation. Moreover, HOXA6 physically interacts with PBX2, and the coexpression of HOXA6 and PBX2 promotes gastric cancer cell migration and invasion. All in all, the above results demonstrate the potential of using HOXA6-PBX2 axis as the therapeutic target for the treatment of GC metastasis.

## MATERIALS AND METHODS

### Cell culture, cell lines and reagents

The human GC cell lines, including MKN28 and MKN45 (Japanese Collection of Research Bioresources Cell Bank, Osaka, Japan); HGC-27 and AGS (ATCC, Rockville, MD); BGC-823, MGC803 and SGC7901 (Beijing Institute of Cancer Research, Beijing, China) were maintained within the RPMI medium 1640 containing 10% fetal bovine serum (FBS) under 37° C and 5% CO_2_ conditions.

Rabbit anti-HOXA6 (18210-1-AP) and anti-HA (20366-1-AP) were purchased from Proteintech (Proteintech, Chicago, USA). Rabbit anti-HOXA6 (HPA004203) and anti-PBX2 (HPA061478) antibodies were purchased from Sigma (Sigma-Aldrich, St. Louis, MO, USA). Mouse anti-PBX2 (sc-377164) was purchased from Santa Cruz (Santa Cruz, CA, USA). Rabbit anti-Flag (14793S) was purchased from Cell Signaling (Cell Signaling, Massachusetts, USA). Rabbit anti-β-tubulin (RM2003) was purchased from Ray Antibody Biotech (Ray Antibody Biotech, Beijing, China).

### Patient specimens and immunohistochemical staining (IHC)

Specimens from twenty-three patients with GC and adjacent nontumor tissues between August 2019 and December 2019 were collected at the Department of Surgery of Nanfang Hospital, Southern Medical University, China. In addition, tissue microarrays (TMAs) for human GC tissue samples and paired non-carcinoma tissue samples were provided by Superchip (Shanghai, China). In this work, altogether 85 GC tissue samples with matched non-carcinoma tissue samples were collected. Our study protocol was approved by the Ethics Committee of Southern Medical University in China (protocol number: NFEC-2017-062).

The GC tissue sample was subjected to formalin fixation and paraffin embedding to conduct immunohistochemical (IHC) staining. Then, Tris-EDTA buffer (pH 9, Dako, Denmark) was added to retrieve the heat-induced epitopes within the microwave. 3% hydrogen peroxide was adopted for blocking endogenous peroxidase activity in the tissue sections, followed by overnight incubation using anti-PBX2 or anti-HOXA6 (1:100) and later incubation with biotin-conjugated anti-rabbit IgG (Dako, Copenhagen, Denmark) combined with the DAB complex, with the normal mouse or rabbit IgG (Sigma) being the isotype reference.

The staining result for HOXA6 and PBX2 in gastric cells was categorized based on their staining intensity, where 0, 1, 2 and 3 respectively represented negative, weak, moderate and intense staining. In this study, The negative or weak staining cells were deemed as those of low expression, while those with moderate and intense staining as high expression cells [40]. For every sample, the mean score assessed by at least 2 reviewers indicated the eventual IHC score. The Nikon Eclipse E600 microscope (Nikon Imaging Japan Inc) was utilized to analyze and photograph the slides.

### Western blot analysis

Samples containing 30 μg of protein each were parted by 4-12% SDS-PAGE, followed by dry transfer to the nitrocellulose membranes (Invitrogen). Then, 5% skimmed milk powder was utilized for blocking the membranes, followed by overnight incubation using the primary antibodies dissolved within the blocking buffer (containing 1× TBST, 3% skimmed milk powder, 0.2% Tween 20) under 4° C. After washing by 1× TBST, the membranes were subjected to further antibody incubation. Then, membranes were washed by 1× PBS that contained 0.2% Tween 20, and ECL chemiluminescence reagents (Pierce) were employed to detect signals. Antibodies against HOXA6, PBX2 and tubulin were used in this study.

### q-RT-PCR assays

In RT-PCR assays, the TRIzol reagent (Invitrogen) was used to isolate total cellular RNA; later, the SuperScript One-step RT-PCR system (Invitrogen) was used to transcribe and amplify the isolated total RNA (2μg) to prepare cDNA. Afterwards, the resultant cDNA was blended with specific primers as well as SYBR Green PCR Master Mix. It was utilized for RT-PCR assay under the following conditions, 20 s under 94° C, 20 s under 55° C as well as 40 s under 72° C for altogether 60 cycles. Specific or control primers were utilized to predict mRNA expression level for twice, the 2^-ΔΔC^t approach was adopted for calculating the mean relative fold change (FC) of mRNA expression, and β-actin was used as the endogenous reference. The oligonucleotide primers for E-cadherin and β-actin mRNA were as previously described [41].

### Transfection and RNA interference

The target siRNA sequences for human HOXA6 were 5'-AGUACACGAGCCCGGUUUATT-3' (no. 1) and 5'-AGGAAAACAAGCUCAUCAATT-3' (no. 2), and those for human PBX2 were 5'-CCGTAACTTCAGCAAACAG-3' (no. 1) and 5'-CATCGAACACTCGGACTAT-3' (no. 2). The control siRNA sequence was 5'-UUCUCCGAACGUGUCACGUTT-3', SiRNAs were purchased from Gene Pharma (Shanghai, China). The Lipofectamine 3000 reagent (Invitrogen) was used in transfection according to specific instructions. Western blotting was conducted to examine the efficiency of transfection.

### Coimmunoprecipitation (Co-IP)

For preparing the target protein precipitate, 3 μg primary antibody and the previously cleared protein agarose bead (Roche, Mannheim, Germany) slurry were used to incubate cell lysates under 4° C for 3 h. Then, the sample was rinsed and analyzed by Western blotting for the sake of detecting the possible protein interactions.

### Determination of the half-lives of the HOXA6 and PBX2 proteins

Zhang et al.’s method [42] was utilized to measure protein half-life. To be specific, cells were exposed to 50μg/ml cycloheximide for specific time periods, harvested through trypsinization, and lysed using anti-PBX2 or anti-HOXA6 antibody for Western blotting assay. Later, the gel documentation system was utilized to measure band intensity, and the readings were presented in the manner of percentage and normalized to the original anti-PBX2 or anti-HOXA6 staining intensity (intensity at the beginning). Later, a curve regarding percentage as a function of time was plotted, and the PBX2 and HOXA6 protein half-life was determined as the time necessary for the degradation of 50% protein.

### Confocal microscopy

Cells that grew on the coverslips were subjected to 4% paraformaldehyde fixation, and then 1% BSA was used to block the nonspecific binding. Afterwards, primary antibodies and TR (Texas Red)- or FITC-labeled secondary antibodies were utilized to incubate the cells in succession. Then, 1 μg/mL Hoechst 33258 was used for counter-staining of nuclei, and nail varnish was used to mount coverslips. Finally, the Zeiss LSM710 confocal microscope was employed to capture the confocal images under the 40× objective.

### EdU incorporation assay

The cell proliferation rate was assessed by EdU incorporation assay. The gastric cancer cells plated in 96-well plates with 2 × 10^3^ cells per well and allowed to adhere overnight. After 2 h of cell incubation using 50 μM EdU under 37° C, cells were subjected to 30 min of 4% formaldehyde fixation. Later, formaldehyde was neutralized using glycine for ensuring the reaction. Afterwards, 0.5% Triton X-100 under ambient temperature were used to permeabilize GC cells; 10 min later, EdU was added to react with 100 μl 1× Apollo^®^ reaction cocktail for 30 min. Finally, nuclei were stained by 100 μl 1× Hoechst. The fluorescence microscope (Nikon, Japan) was employed for cell visualization.

### Cell migration and transwell invasion assays

The AGS and BCG-823 cell lines after transfection were grown into the 6-well plates until confluence in scratch wound assay, and a pipette tip was then used to make a wound. Cells were subjected to incubation under 37° C and 5% CO_2_ conditions, then the closure of wound was monitored for 48-72 h and photos were taken. All assays were carried out for three times.

In Transwell invasion assay, the Matrigel basement membrane matrix (Becton Dickinson Labware) was prepared into the 24-well Transwell chambers according to specific instructions. The cell suspension was prepared in RPMI and put into the upper chamber at the density of 1×10^5^ cells/well. Later, 5% FBS containing RPMI was added to the lower chamber as the chemoattractant. Cells were cultured for 24 h, a cotton swab was used to wipe the upper surface of the Transwell chamber, and 0.2% crystal violet was utilized to stain the inserts. Finally, the number of invading cells within 3 fields of view (FOV) in each insert was calculated by the light microscope, and the average value was calculated.

### Immunofluorescence

For F-actin staining, cells were subjected to 1 h of 4% paraformaldehyde fixation, 5 min of 0.2% Triton X-100 incubation, 20 min of 3% bovine serum albumin (BSA) incubation, and 1h of rhodamine-labeled phalloidin (1:100) incubation, which purchased from Molecular Probes, Eugene, Oregon, USA. Thereafter, Vectashield (Vector Laboratories, Burlingame, California, USA) was used to mount each slide, and the fluorescence microscope was employed for visualization. Later, 1 μg/mL Hoechst 33258 was used to stain nuclei, and the fluorescence microscope was adopted for cell analysis.

### Plasmid construction and lentiviral preparation

RT-PCR amplification was carried out to prepare normal human cDNA with related to the full-length PBX2 or HOXA6. Thereafter, the PCR products obtained were further cloned to pENTER-FLAG or pENTER-HA mammalian expression vector (Vector, Vigene Biosciences, Rockville, MD, USA). Next, stable GC cell lines with ectopic HOXA6 or PBX2 expression were established using Lipofectamine TM 3000.

Lentiviruses expressing EGFP/HOXA6 (LV-HOXA6) were synthesized by GeneChem (Shanghai, China) by the use of Ubi-MCS-3FLAG-CBh-gcGFP-IRES-puromycin vector, whereas the corresponding empty vector served as internal reference (Shanghai GeneChem Co, Ltd, China).

The double-stranded oligonucleotides that encoded human HOXA6-vshRNA (NM_024014: HOXA6 shRNA 2: CCGGAGGAAAACAAGCUCAUCAATCAAGAGUUGAUGAGCUUGUUUUGGUTTTTTG) or PBX2-vshRNA (NM_002586: PBX2 shRNA 2: CCGGCAUCGAACACUCGGACUAUUCAAGAGGUAGCUUGUGAGCCUGAUAUUUUG) were annealed and inserted into the U6-MCS-Ubiquitin-Cherry-IRES-puromycin short hairpin RNA (shRNA) expression vector. The selective overexpression or knockdown cells were applied in later analysis.

### Cycloheximide treatment

For determining whether endogenous PBX2 and HOXA6 proteins were stable, the PBX2 plasmid, HOXA6 plasmid or empty plasmid was transfected into cells in the presence of 50 μg/ml cycloheximide for specific time periods. Then, BCA assay was conducted to determine total protein content within cell lysates. After separation by SDS-PAGE, the endogenous HOXA6 and PBX2 protein contents in lysates were measured through Western blotting.

### Animal experiments

Each animal procedure was performed following the Southern Medical University animal care guidelines. Besides, animals were handled according to the Institutional guidelines (authorization protocol number: NFEC-2017-062). Besides, animal suffering was minimized, the animal number utilized was reduced, and candidate alternatives were adopted for *in vivo* assays. The 0.25% trypsin/0.02% EDTA solution (w/v) was added to collect AGS cells at exponential phase. Later, trypan blue exclusion was adopted to determine the viability of AGS cells, and just single-cell suspension with over 95% viable cells were utilized. For evaluating the metastasis ability of tumor cells *in vivo*, BALB/Cnu/nu nude mice were injected through the tail vein with 4 × 10^5^ lentivirus-vector, lentivirus-HOXA6 shRNA or lentivirus-HOXA6, and lentivirus-HOXA6-PBX2 shRNA-transfected cells per mouse. All mice were sacrificed at 4 weeks following injection to remove the lungs and other organs for assessment by the InVivoF Imaging System (Kodak). Then, the collected tissues were subjected to 10% buffered formalin fixation, gradient alcohol immersion, as well as paraffin embedding. Each tissue section was acquired by the routine approach, followed by HE and IHC staining along with qRT-PCR analysis.

### Statistical analysis

All the Statistical analysis was principally processed by SPSS20.0 software (Chicago, IL). Values were expressed in the manner of mean ± SD. Pearson’s χ2 test was operated to analyze the association between the expression of HOXA6 and clinicopathologic features. Kaplan-Meier test was applied in calculating survival rate, while log-rank test was utilized in examining different survival rates of both groups. The correlation of HOXA6 level with PBX2 expression in tissue was assessed by correlation analysis. A two-tailed p<0.05 was deemed as statistically significant.

## Supplementary Material

Supplementary Figures
