# Supplementary material for: Coexpression of HOXA6 and PBX2 promotes metastasis in gastric cancer
Source: Aging (Albany NY). 2021 Feb 1;13(5):6606–24. doi: 10.18632/aging.202426 (PMC7993744; doi:10.18632/aging.202426)
Supplement: Supplementary Figures [file aging-13-202426-s001.pdf]

SUPPLEMENTARY FIGURES

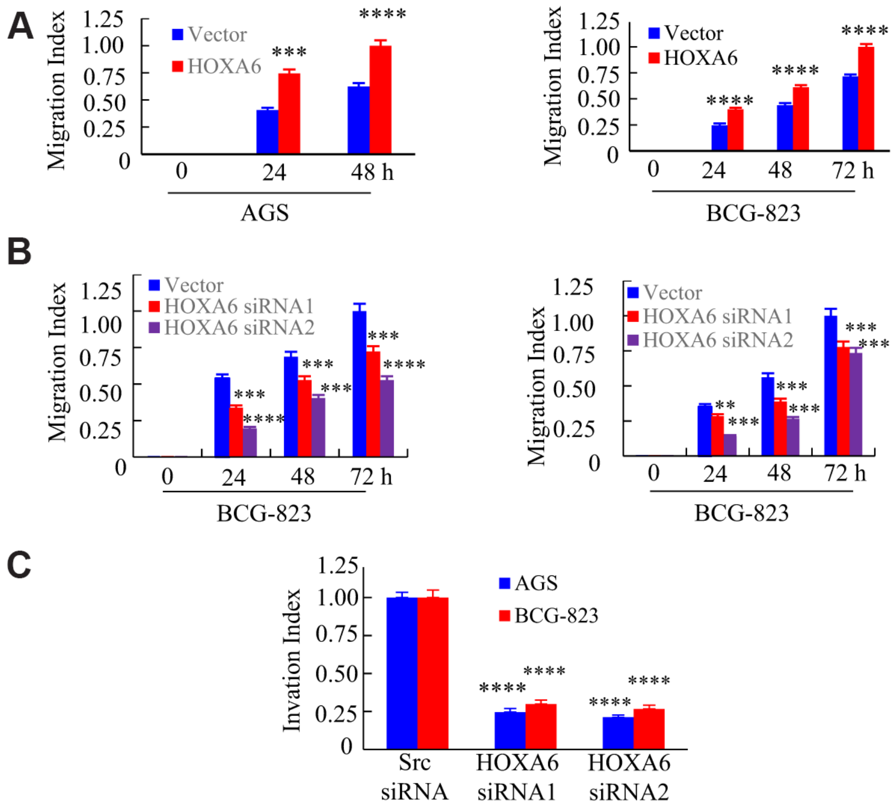

Supplementary Figure 1. HOXA6 on invasion and migration in GC cells *in vitro*. (**A**, **B**) Histogram showing the percentage of wound closure. (**, P <0.05; ***, P<0.01; ****, P<0.001 compared with 0 h). (**C**) Invading cells were counted by microscopy. ****, P<0.001. Data are repeated at least three independent experiments with similar results on average.

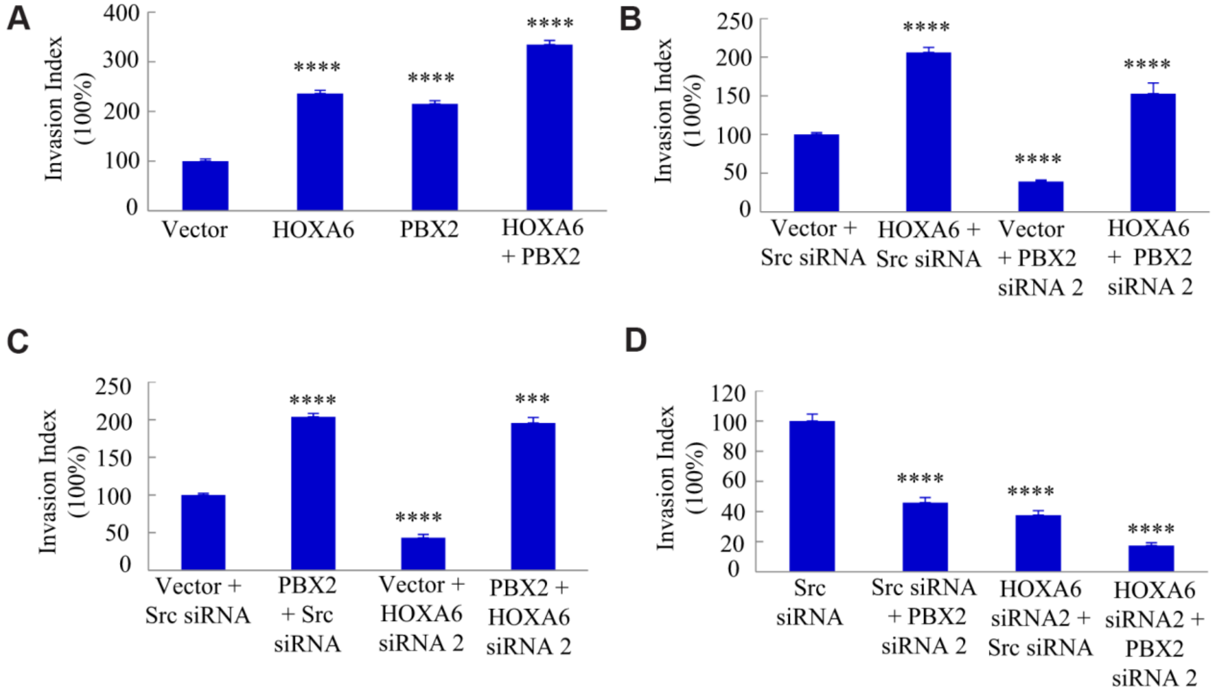

Supplementary Figure 2. GC cells were evaluated by Transwell invasion assay. (**A**–**D**) Invading cells were counted by microscopy. ***, P<0.01, ****, P<0.001.
